# Associations between loneliness severity and depression and suicidal ideation among older adults during the COVID-19 pandemic

**DOI:** 10.1080/13607863.2026.2612734

**Published:** 2026-01-23

**Authors:** Kevin H. Yang, Wayne Kepner, Nora Satybaldiyeva, Jaclyn Bergstrom, Alison A. Moore, Ellen E. Lee

**Affiliations:** a Department of Psychiatry, University of California San Diego School of Medicine, San Diego, CA, USA; b Department of Psychiatry & Behavioral Sciences, Stanford University School of Medicine, Palo Alto, CA, USA; c Stanford Prevention Research Center, Stanford University School of Medicine, Palo Alto, CA, USA; d Division of Geriatrics, Gerontology and Palliative Care, University of California San Diego School of Medicine, San Diego, CA, USA; e Desert-Pacific Mental Illness Research Education and Clinical Center, Veterans Affairs San Diego Healthcare System, San Diego, CA, USA

**Keywords:** Loneliness, depression, suicidal ideation, older adults, all of us research program

## Abstract

**Objectives:**

The COVID-19 pandemic has exacerbated loneliness among older adults. This study evaluates the relationship between loneliness, depression, and suicidal ideation in this population during the early pandemic by utilizing data from the All of Us (AoU) Research Program.

**Method:**

We analyzed cross-sectional data from 17,084 individuals aged ≥65 enrolled in the AoU Research Program who completed a COVID-19 related survey in May 2020. Participants were categorized into loneliness quartiles based on their UCLA Loneliness Scale Short Form-8 scores. Descriptive statistics and chi-square tests assessed demographic differences. Poisson regression models with robust standard errors tested associations between loneliness quartiles and both depression and suicidal ideation, adjusting for age, gender, race, ethnicity, education, marital status, employment status, housing status, health insurance status, and social support.

**Results:**

Among 17,084 participants (mean age 72.0 years [SD 5.3], 55% female, 89% White), we found a clear stepwise pattern: as loneliness increased, so did the risk of depression and suicidal ideation. Compared to the lowest loneliness quartile, those in the highest loneliness quartile were approximately 25 times more likely to experience moderate-to-severe depression (adjusted relative risk [aRR] = 25.09, 95% CI: 16.63–37.84) and 44 times more likely to report suicidal ideation (aRR = 44.31, 95% CI: 19.69-99.71). Intermediate loneliness levels (quartiles 2–3) also showed elevated risks (depression aRRs: 2.45, 7.33; suicidal ideation aRRs: 4.03, 11.87).

**Conclusion:**

Increasing levels of loneliness were associated with greater risk of depression and suicidal ideation among older adults during the early pandemic, underscoring the need for targeted loneliness interventions for this vulnerable population.

## Introduction

The COVID-19 pandemic had profound impacts on mental health across all age groups, with older adults experiencing particularly severe challenges (COVID-19 Mental Disorders Collaborators, 2021; Zaninotto et al., 2022). Enforced social distancing, disruptions in non-essential activities, and limitations on in-person gatherings created unprecedented conditions of restricted social contact (MacLeod et al., 2021; Morlett Paredes et al., 2021; O’Sullivan et al., 2021; Wu, 2020). Due to their higher risk of COVID-related hospitalizations, older adults were more likely to practice strict social distancing (MacLeod et al., 2021). In particular, community-dwelling older adults were significantly impacted by local stay-at-home orders which banned visits for most nursing home and senior community residents (Wu, 2020). Studies have shown that these pandemic-related restrictions increased social isolation (the objective condition of having few social connections and infrequent social contact), which may have intensified loneliness (the subjective feeling of distress from unmet social needs) and reduced perceived social support (the availability of resources and assistance from others) among older adults (Defrancesco et al., 2024; Holt-Lunstad, 2022; Kirkland et al., 2023).

Loneliness, defined as the discrepancy between a person’s desired and existing social relationships, disproportionately affects older adults, even under normal circumstances. Over 40% of those aged ≥60 report feeling lonely (Luhmann et al., 2023; Social Isolation & Loneliness in Older Adults, 2020). The older adult population faces unique challenges that can make it more difficult to counteract the negative effects of loneliness. Examples of these challenges include death of family members, friends, and partners, physical and cognitive decline, comorbidities, frailty, functional limitations, and changes in living arrangements or family structure (Luhmann et al., 2023). Loneliness is also a major risk factor for declining mental and physical health (Donovan & Blazer, 2020). Additionally, older adults are increasingly choosing to live alone, which is a demographic trend that may increase feelings of loneliness (Pickering et al., 2023). Depression and loneliness have a bidirectional relationship in which each condition potentially exacerbates the other (Leigh-Hunt et al., 2017).

Research prior to the COVID-19 pandemic suggests a link between loneliness and an increased risk of depression (persistent feelings of sadness or loss of interest that interfere with daily functioning) (Courtin & Knapp, 2017; Leigh-Hunt et al., 2017; Park et al., 2020; Solmi et al., 2020; Wang et al., 2018) and suicidal ideation (thoughts about ending one’s own life) (Leigh-Hunt et al., 2017; Park et al., 2020; Solmi et al., 2020). Furthermore, depression rates among older adults have already been increasing in the decade leading up to the pandemic (2010–2019) (Yang et al., 2022), which underscores the need for further research into the mental health impacts of loneliness. The relationship between loneliness and mental health became particularly important during the COVID-19 pandemic given widespread enforced isolation and intensified loneliness. In 2023, reflecting the ongoing public health significance of this issue, the United States (US) Surgeon General identified loneliness as a significant public health crisis (Office of the Surgeon General & Public Health Service, 2023). This designation highlights the need for research into the mental health impacts of loneliness among older adults in the context of the COVID-19 pandemic.

While emerging research has documented mental health deterioration among older adults during the COVID-19 pandemic, existing studies have been limited by several factors, including small sample sizes, lack of diversity in study populations, lack of examination across sociodemographic subgroups (e.g. age, gender, marital status, and education), focus on populations outside the US, and failure to consider varying degrees of loneliness (Hung et al., 2024; Kotwal et al., 2021; Krendl & Perry, 2021; Liu et al., 2023; Macalli et al., 2022; O’Shea et al., 2021; Tachikawa et al., 2023; Wister et al., 2023). Given these gaps, additional research is needed to examine how the severity of loneliness affects depression and suicidal ideation among older adults aged ≥65 in the US during the COVID-19 pandemic.

The present study utilizes data from the All of Us Research Program, which had enrolled over 509,000 participants across the US at the time of our analysis. Our study aims to quantify the associations between quartiles of loneliness and both depression and suicidal ideation for adults aged ≥65 during the COVID-19 pandemic. By doing so, we will elucidate the extent to which increasing levels of loneliness affect older adults’ self-reported symptoms of depression and suicidal ideation. While previous studies have established a general association between loneliness and mental health outcomes, our study makes several unique contributions: (1) quantifying the strength of associations across different levels of loneliness severity; (2) measuring these associations during an unprecedented period of enforced isolation, providing insights for future public health emergencies; and (3) identifying potential threshold levels of loneliness that may warrant clinical intervention. These findings can be used to inform age-appropriate and targeted interventions to potentially mitigate the negative mental health effects of the COVID-19 pandemic and may guide clinical decision making in mental health screenings for older adults.

## Methods

### Data source

We analyzed cross-sectional data from the National Institutes of Health (NIH) All of Us Research Program, which compiles surveys, electronic health record data, and physical measurements from participants. At the time of our analysis, the program had enrolled over 509,000 participants, with more than 80% of participants from communities traditionally underrepresented in biomedical research, which the program broadly defines as meeting any of several categories: racial or ethnic minority, age 65 and older, sexual and gender minority, individuals with low income or education attainment, rural residents, or those with disabilities. Participants can sign up directly through the NIH All of Us Research Program website or through participating health care provider organizations. They receive a one-time compensation of $25 in the form of cash, gift card, or electronic voucher if asked to give physical measurements or specimens. The All of Us Research Program is approved by the NIH Institutional Review Board (IRB). Participants sign an informed consent authorizing the collection of their data. Researchers with authorization can access de-identified participant data. Due to the secondary nature of this analysis and no direct involvement of human subjects, the University of California San Diego IRB exempted this study from review.

### Study sample

Our study sample consisted of adults aged ≥65 who had completed the COVID-19 Participant Experience (COPE) survey in May 2020 (*N* = 17,084). This brief online survey was distributed to a subset of participants from the All of Us Research Program to understand the impact of the COVID-19 pandemic. It included questions on COVID-19 symptoms, social distancing practices, general well-being, physical and mental health, and coping strategies.

### Loneliness

Loneliness was assessed using the UCLA Loneliness Short Form-8, an 8-item checklist that employs a 4-point Likert scale to measure current feelings of loneliness (Hays & DiMatteo, 1987). Participants’ responses were aggregated to yield scores between 8 and 32, with higher scores reflecting more severe loneliness. In the absence of established cut-off points for this scale, we opted to categorize loneliness into quartiles (from ‘least lonely’ (1st quartile) to ‘most lonely’ (4^th^ quartile)), an analytic approach supported by previous studies (Harrison et al., 2025; Kantor & Kantor, 2020).

For the UCLA Loneliness Short Form-8, the Kaiser–Meyer–Olkin value indicated excellent sampling adequacy (KMO = 0.87), and Bartlett’s Test of Sphericity was significant (*p* < 0.001), confirming suitability for factor analysis. Factor analysis supported a single-factor solution, consistent with the original scale (Ausín et al., 2019; Zhong et al., 2024). Factor loadings ranged from 0.54 to 0.75 for seven items, with one item showing a low loading. The factor accounted for 39.5% of total variance. Skewness values ranged from 0.44 to 1.74, indicating slight positive skew which is expected given most participants are not highly lonely. Kurtosis values ranged from −1.1 to 2.5, indicating mild leptokurtosis common in psychosocial scales.

### Depression and suicidal ideation

Depression was assessed using the Patient Health Questionnaire (PHQ-9), a 9-item checklist reflecting the Diagnostic and Statistical Manual of Mental Disorders (DSM-IV) criteria for depressive symptoms over the preceding two weeks, scored on a 4-point Likert scale (American Psychiatric Association, 1994). A composite score, ranging from 0 to 27, was computed by summing the responses to all items, with higher scores indicative of greater depression severity. Following standard PHQ-9 scoring criteria, scores of 10 and above were classified as moderate-to-severe depression (Manea et al., 2012). Suicidal ideation was assessed through the ninth question of the PHQ-9, which directly asked if participants had ‘Thoughts that you would be better off dead or hurting yourself in some way’ in the past 2 weeks. Consistent with previous studies (Xu et al., 2024), responses were dichotomized as affirmative (i.e. those who answered ‘some’, ‘often’, or ‘nearly all of the time’) or negative (i.e. those who answered ‘not at all’).

For the PHQ-9, the Kaiser-Meyer-Olkin value indicated excellent sampling adequacy (KMO = 0.90), and Bartlett’s Test of Sphericity was significant (*p* < 0.001), confirming suitability for factor analysis. Factor analysis supported a single-factor solution, consistent with the original scale. Factor loadings ranged from 0.42 to 0.79 for all items, with strongest loadings on core depressive symptoms (e.g. depressed mood, anhedonia, and fatigue) and modest loadings for the sleep and suicidal ideation terms, consistent with prior psychometric reports (Bianchi et al., 2022; Shaff et al., 2023). The factor accounted for 40.6% of total variance. Skewness values ranged from 1.12 to 7.30 and kurtosis values ranged from 0.56 to 63.63, indicating expected non-normality, reflecting low depressive symptom burden in this community sample.

### Social support

Perceived social support over the previous month was measured using 10 items from the RAND Medical Outcomes Study Social Support Survey Instrument (Sherbourne & Stewart, 1991). These items were rated on a 5-point Likert scale, with total scores ranging from 10 to 50. Higher scores signified greater perceived social support.

### Sociodemographic characteristics

Sociodemographic data were categorized as follows: age (continuous), gender (female, male, other), race (White, Black, Asian, none indicated, other), ethnicity (Hispanic, Non-Hispanic), educational attainment (less than high school, high school, some college, college graduate, not answered), marital status (married, not married), employment status (employed, unemployed, and retired), housing status (housed [i.e. studio, one-bedroom apartment, two-bedroom apartment, three-bedroom (or more) apartment, townhouse, free-standing house], nursing home, or other), and health insurance status (insured and uninsured).

### Statistical analysis

We employed descriptive statistics to characterize the overall study sample and subgroups defined by loneliness quartiles. We reported frequencies with percentages for categorical variables and means with standard deviations for continuous variables. Differences in sociodemographic characteristics, depression, suicidal ideation, and social support across loneliness quartiles were analyzed using chi-squared tests for categorical variables and ANOVA for continuous variables. Poisson regression with robust standard errors was used to estimate risk ratios for associations between loneliness quartiles and both depression and suicidal ideation. We chose this approach because odds ratios from logistic regression can overestimate associations when outcome prevalences vary across exposure groups. In our sample, depression prevalence ranged from 0.7% in the lowest loneliness quartile to 22.8% in the highest quartile, and suicidal ideation prevalence ranged from 0.2% to 10.6%. With such variation, odds ratios would overstate the magnitude of associations. Relative risks thus provide a more accurate and interpretable measure of association, showing how many times more likely individuals in higher loneliness groups are to experience depression or suicidal ideation compared to the lowest loneliness group (Zou, 2004). For example, a relative risk of 2.0 means an individual is twice as likely to experience the outcome. These models were adjusted for age, gender, race, ethnicity, educational attainment, marital status, employment status, housing status, health insurance status, and social support rating. We reported adjusted relative risks (aRRs) with 95% confidence intervals (CIs) and corresponding *p*-values.

To assess potential common method bias, we conducted Harman’s Single-Factor Test to evaluate survey items from our primary exposure and outcome measures (UCLA Loneliness Short Form-8 and PHQ-9). An unrotated exploratory factor analysis revealed that the first factor accounted for 19% of the total variance, which is well below the convention 40% threshold used to indicate substantial common method bias. Moreover, loneliness and depression items loaded distinctly, with loneliness items demonstrating moderate loadings (0.53–0.75) and PHQ-9 items exhibiting negligible loadings (≈0.00–0.06) on the first factor, supporting construct separability. These findings suggest that common method bias is unlikely to pose a meaningful threat to the validity of our results.

For all analyses, statistical significance was defined as *p* < 0.05. All analyses were performed using Python within the All of Us Workbench environment.

## Results

Table 1 delineates the sociodemographic and mental health characteristics of the study participants. Among the 17,084 participants (mean [SD] age 72.0 years [5.3]), 55.0% were female, and 89.8% self-identified as White. A small percentage identified as Black (2.9%), Asian (1.2%), or Hispanic (2.1%). Most participants were married (66.9%), had attained a college degree (72.0%), were retired (70.3%), were housed (96.3%), and had health insurance coverage (99.2%). In this sample, 7.8% met criteria for moderate-to-severe depression, and 3.5% reported suicidal ideation.

Table 2 stratifies participants’ sociodemographic and mental health characteristics by loneliness quartiles. Compared to individuals in the lowest quartile of loneliness, those in the highest quartile of loneliness were younger, more likely to be female, and reported lower levels of social support (all *p*s <0.001). They were also less likely to be married, employed, have a college degree, live in private housing, or have health insurance coverage (all *p*s <0.05). No significant racial or ethnic differences were observed across the loneliness quartiles. Those in the highest quartile of loneliness also had markedly higher rates of both moderate-to-severe depression (22.8%) and suicidal ideation (10.6%) compared to those in lower quartiles (all *p*s < 0.001).

Tables 3 and 4 present the results of univariable and multivariable Poisson regression analyses, which were used to examine the unadjusted and adjusted associations between loneliness severity and each mental health outcome (depression and suicidal ideation) separately. We found a clear stepwise pattern: older adults with higher levels of loneliness were progressively more likely to experience both depression and suicidal ideation. Compared to the first (lowest) loneliness quartile, older adults in the second, third, and fourth quartiles were more likely to experience moderate-to-severe depression, with aRRs of 2.45 (95% CI: 1.55–3.89), 7.33 (95% CI: 4.81–11.17), and 25.09 (95% CI: 16.63–37.84), respectively. Similarly, the association between loneliness and suicidal ideation increased in a stepwise fashion with higher quartiles of loneliness; individuals in the second, third, and fourth quartiles of loneliness had aRRs of 4.03 (95% CI: 1.67–9.70), 11.87 (95% CI: 5.19–27.14), and 44.31 (95% CI: 19.69–99.71) for suicidal ideation, respectively, compared to those in the first quartile.

## Discussion

We identified a strong, positive association between loneliness and increased levels of depression and suicidal ideation among older adults during the COVID-19 pandemic. Notably, these associations showed a clear stepwise pattern; those in the highest quartile for loneliness were approximately 25 times more likely to suffer from depression and 44 times more likely to have suicidal thoughts, compared to their counterparts in the lowest quartile. To our knowledge, this is the first study to report associations of this magnitude, highlighting the severity of the loneliness epidemic among older adults in the US during the COVID-19 pandemic. These findings underscore the need for mental health screening and the development of interventions specifically targeting loneliness in this vulnerable population.

Our findings align with prior research connecting loneliness among older adults to depression (Courtin & Knapp, 2017; Kotwal et al., 2021; Krendl & Perry, 2021; Leigh-Hunt et al., 2017; Liu et al., 2023; O’Shea et al., 2021; Park et al., 2020; Solmi et al., 2020; Wang et al., 2018; Wister et al., 2023). Building upon this knowledge, we demonstrated, through both unadjusted and adjusted analyses, a graded relationship between the severity of loneliness and the prevalence of depression. The underlying mechanisms for these associations are multi-faceted. First, loneliness may be a risk factor for depression and other mental health symptoms. For instance, loneliness may lead to negative self-perception and rumination, which may contribute to depressive symptoms (Mushtaq et al., 2014). Lonely individuals may also resort to unhealthy coping strategies, such as unhealthy substance use, to mitigate feelings of loneliness, which could exacerbate depression and other mental health symptoms (Ingram et al., 2020; Mushtaq et al., 2014). Individuals experiencing loneliness may exhibit elevated cortisol levels, which can also adversely impact mental health outcomes (Qin et al., 2016). Second, depression may also lead to loneliness. Individuals with depression may experience decreased motivation, withdraw from social activities and interactions, or have communication challenges, which could result in decreased social connections and feelings of loneliness (Mushtaq et al., 2014). Those experiencing depression may also face stigma from family and friends, leading them to isolate themselves (Prizeman et al., 2023). Other factors, such as comorbid cognitive impairment, chronic medical conditions, disability, childhood trauma, and personality disorders, may also contribute to the complex relationship between loneliness and depression (Luhmann et al., 2023). Further studies are needed to examine the impact of these factors on the relationship between loneliness and depression.

We also found significant associations between the severity of loneliness and suicidal ideation among older adults during the COVID-19 pandemic. Older adults in the highest loneliness quartile had 44 times higher risk of suicidal ideation compared to those in the lowest quartile, which greatly exceeds the associations (typically odds ratios of 4–6) reported in previous studies during the pandemic. However, these studies employed different scales for measuring loneliness and suicidal ideation than the present study and had notable differences in their sample characteristics, such as age and depression levels (Hung et al., 2024; Macalli et al., 2022; Tachikawa et al., 2023). Macalli et al. (2022) used cross-sectional data of 1,913 college students in France and found that individuals with high levels of loneliness (those who scored ≥7 on a single-item question ‘In the last seven days, on a scale of 0–10 [0 not at all, 10 = totally], how lonely do you feel?’) had an aOR of 4.34 for reporting suicidal ideation over the last seven days. Hung et al. (2024) used cross-sectional data of 8460 participants in Taiwan with a wide age range (15–24, 25–64, and ≥65) and found that individuals with loneliness (defined *via* the question ‘Do you often feel lonely or isolated’ and dichotomized as ‘lonely’ and ‘not lonely’) had an aOR of 4.9 for lifetime suicidal ideation compared to individuals without feelings of loneliness. These studies used single-item questions to assess loneliness, which may lead to under-reporting or gender differences in reporting, whereas our study employed the UCLA Loneliness Short Form-8, which does not explicitly use the word ‘loneliness’. In the present study, even older adults in the second quartile of loneliness were almost 4 times more likely to report suicidal ideation, with exponential increases as loneliness severity increased to higher quartiles. While our findings cannot establish causality, these findings from early in the COVID-19 pandemic demonstrate particularly strong associations between loneliness and suicidal ideation in older adults. These associations may reflect the complex interplay between loneliness, pandemic-related restrictions that increased social isolation measures, and mental health during this period. Our results emphasize the critical need for screening and suicide prevention strategies prioritizing older adults suffering from severe loneliness and highlight the importance of loneliness as a risk factor for suicidal ideation specifically in older adults.

Our findings highlight the urgent need to develop, test, and implement evidence-based interventions specifically tailored to address loneliness among older adults. Social media and digital health technology have shown efficacy in numerous intervention studies in older adults and could be particularly relevant in future situations where social distancing measures are enforced (Fakoya et al., 2020; O’Rourke et al., 2018; Wister et al., 2021). Other evidenced-based interventions and strategies to combat loneliness include: increased personal contact, activity groups and discussions, animal-assisted therapy, psychotherapy, volunteering opportunities, and skills training courses, such as resilience and self-kindness (Banks & Banks, 2002; da Silva-Sauer et al., 2025; Fakoya et al., 2020; Hoang et al., 2022; Kim et al., 2025; Shekelle et al., 2024; Solomonov, 2023; Van Orden et al., 2013, 2021; Warner et al., 2024). However, the evidence for these loneliness interventions vary in terms of their generalizability and efficacy, which is important to consider for the unique circumstances surrounding the COVID-19 pandemic (Hoang et al., 2022; Shekelle et al., 2024). As geriatric health professionals, public health officials, and policymakers aim to address loneliness among the older adult population, they must carefully consider and build upon these existing intervention approaches while recognizing the need for more tailored and targeted interventions.

### Limitations

Our findings should be interpreted in the context of several limitations. First, the cross-sectional design of our study precludes establishing causality or temporality. Second, while our sample is large, it lacks demographic diversity with participants being predominantly White and college-educated, which may be partly due to selection factors such as computer and internet access to complete the online COPE survey, and thus may not be representative of the broader US population. Third, because we relied on the UCLA Loneliness Short Form-8, which lacks established cut-off scores for high levels of loneliness, we categorized levels of loneliness by quartiles, consistent with previous studies (Harrison et al., 2025; Kantor & Kantor, 2020). Future research may benefit from incorporating additional scales of loneliness with validated cut-off scores such as the full 20 question UCLA Loneliness Scale (Russell, 1996). Fourth, suicidal ideation was assessed using only item 9 of the PHQ-9. While this single-item measure is widely used and is a moderate predictor of future suicidality (Rossom et al., 2017; Simon et al., 2016), it does not capture the severity, frequency, or intent of suicidal thoughts. Future studies would benefit from using validated multi-item instruments such as the Columbia-Suicide Severity Rating Scale (C-SSRS) to provide a more detailed assessment of suicidal ideation and behavior. Fifth, while data were collected in May 2020, this early pandemic timing represents a unique natural experiment that cannot be replicated. The fundamental relationship between loneliness severity and mental health outcomes likely reflects psychological mechanisms that extend beyond the specific pandemic context, with relevance for future public health emergencies. Nevertheless, the unique timing of our study during the COVID-19 pandemic provides a valuable snapshot of the relationship between loneliness and key mental health outcomes among older adults during this time period. Finally, there may be unmeasured factors, such as the extent of lockdown measures, that could have influenced the observed associations.

### Conclusion

Loneliness among older adults in the US has significant public health implications. Our study quantified the association between the severity of loneliness (i.e. quartiles) and both depression and suicidal ideation during the COVID-19 pandemic. Our findings suggest a significant progressive relationship between these variables, with those in the highest loneliness quartile being approximately 25 times more likely to experience moderate-to-severe depression and 44 times more likely to report suicidal ideation compared to those in the lowest loneliness quartile. These findings underscore the urgent need for targeted interventions for older adults affected by loneliness.

The COVID-19 pandemic created unprecedented conditions that intensified loneliness, but also provided critical insights for future pandemic preparedness. Our results demonstrate that during public health emergencies requiring isolation measures, ongoing monitoring, screening, and intervention for loneliness should be prioritized, particularly for vulnerable populations such as older adults. Social media and digital health technologies may be particularly valuable tools for maintaining social connections and delivering interventions when in-person contact is restricted, though further research is needed to establish their effectiveness in reducing loneliness and associated mental health risks during public health emergencies. Future longitudinal studies are needed to better understand how loneliness influences depression and suicidal ideation in older adults over time. Addressing loneliness and its mental health consequences among older adults should remain a top public health priority, and further research is needed on this critical issue.

## Figures and Tables

**Table 1.** Sample characteristics of older adults (≥65 years) in the All of Us Research Program.

		Overall
*N*		17,084
Age (yrs), mean (SD)		72.0 (5.3)
Gender, *n* (%)	Female	9388 (55.0)
	Male	7361 (43.1)
	Other	335 (2.0)
Race, *n* (%)	Asian	203 (1.2)
	Black	490 (2.9)
	White	15,338 (89.8)
	None Indicated	720 (4.2)
	None of these	333 (1.9)
Hispanic ethnicity, *n* (%)	Yes	360 (2.1)
Education, *n* (%)	<High School	42 (0.2)
	High School	972 (5.7)
	Some College	3467 (20.3)
	College Graduate	12,309 (72.0)
	Not Answered	294 (1.7)
Married, *n* (%)	Yes	11,429 (66.9)
Employed, *n* (%)	Yes	3494 (20.5)
	No	1578 (9.2)
	Retired	12,012 (70.3)
Housing status, *n* (%)	Housed	16,450 (96.3)
	Nursing home	15 (0.1)
	Other	619 (3.6)
Health insurance, *n* (%)	Yes	16,950 (99.2)
Social support score, mean (SD)		39.5 (10.6)
PHQ-9 depression score ≥10, *n* (%)	Yes	1333 (7.8)
Suicidal ideation, *n* (%)	Yes	592 (3.5)
UCLA loneliness, *n*	Quartile 1: 08–10	3869 (22.6)
	Quartile 2: 11–13	4477 (26.2)
	Quartile 3: 14–17	4379 (25.6)
	Quartile 4: 18–32	4359 (25 5)

Note: Loneliness quartiles based on UCLA Loneliness Scale Short Form-8 Scores: Q1 = least lonely (scores 8-10), Q2 (scores 11-13), Q3 (scores 14-17), Q4 = most lonely (scores 18-32).

Abbreviations: SD: standard deviation; PHQ-9: Patient Health Questionnaire-9.

**Table 2.** Sample characteristics of older adults (≥65 years) in the All of Us Research Program by loneliness quartile.

		Quartile 1 (score 8–10)	Quartile 2 (score 11–13)	Quartile 3 (score 14–17)	Quartile 4 (score 18–32)	*p*-Value
*N*		3869	4477	4379	4359	
Age (yrs), mean (SD)		72.6 (5.3)	72.2 (5.3)	72.1 (5.3)	71.3 (5.1)	<0.001
Gender, *n* (%)	Female	1800 (46.5)	2322 (51.9)	2567 (58.6)	2699 (61.9)	<0.001
	Male	2008 (51.9)	2076 (46.4)	1721 (39.3)	1556 (35.7)	
	Other	61 (1.6)	79 (1.8)	91 (2.1)	104 (2.4)	
Race, *n* (%)	Asian	41 (1.1)	63 (1.4)	53 (1.2)	46 (1.1)	0.102
	Black	116 (3.0)	112 (2.5)	125 (2.9)	137 (3.1)	
	White	3474 (89.8)	4058 (90.6)	3939 (90.0)	3867 (88.7)	
	None Indicated	169 (4.4)	163 (3.6)	178 (4.1)	210 (4.8)	
	None of these	69 (1.8)	81 (1.8)	84 (1.9)	99 (2.3)	
Hispanic ethnicity, *n* (%)	Yes	87 (2.2)	84 (1.9)	88 (2.0)	101 (2.3)	0.446
Education, *n* (%)	<High School	5 (0.1)	9 (0.2)	11 (0.3)	17 (0.4)	<0.001
	High School	242 (6.3)	226 (5.0)	215 (4.9)	289 (6.6)	
	Some College	760 (19.6)	876 (19.6)	873 (19.9)	958 (22.0)	
	College Graduate	2798 (72.3)	3302 (73.8)	3193 (72.9)	3016 (69.2)	
	Not Answered	64 (1.7)	64 (1.4)	87 (2.0)	79 (1.8)	
Married, *n* (%)	Yes	3252 (84.1)	3409 (76.1)	2886 (65.9)	1882 (43.2)	<0.001
Employed, *n* (%)	Yes	835 (21.6)	918 (20.5)	879 (20.1)	862 (19.8)	<0.001
	No	264 (6.8)	358 (8.0)	403 (9.2)	553 (12.7)	
	Retired	2,770 (71.6)	3,201 (71.5)	3,097 (70.7)	2,944 (67.5)	
Housing status, *n* (%)	Housed	3747 (96.8)	4331 (96.7)	4210 (96.1)	4162 (95.5)	<0.001
	Nursing home	0 (0)	3 (0.1)	2 (0.0)	10 (0.2)	
	Other	122 (3.2)	143 (3.2)	167 (3.8)	187 (4.3)	
Health insurance, *n* (%)	Yes	3847 (99.4)	4452 (99.4)	4337 (99.0)	4314 (99.0)	0.015
Social support score, mean (SD)		45.4 (7.6)	43.2 (8.1)	39.5 (9.2)	30.5 (10.5)	<0.001
PHQ-9 depression score ≥10, *n* (%)	Yes	27 (0.7)	76 (1.7)	236 (5.4)	994 (22.8)	<0.001
Suicidal Ideation, *n* (%)	Yes	7 (0.2)	30 (0.7)	93 (2.1)	462 (10.6)	<0.001

Note: Loneliness quartiles based on UCLA Loneliness Scale Short Form-8 Scores: Q1 = least lonely (scores 8–10), Q2 (scores 11–13), Q3 (scores 14–17), Q4 = most lonely (scores 18–32).

Abbreviations: SD: standard deviation; PHQ-9: Patient Health Questionnaire-9.

**Table 3.** Univariable and multivariable associations of severity of loneliness with depression.

	RR (95% CI)	*p*-value
Unadjusted		
Loneliness Q1: 8–10	Ref	
Loneliness Q2: 11–13	2.43 (1.57–3.77)	0.0001
Loneliness Q3: 14–17	7.72 (5.20–11.47)	<0.0001
Loneliness Q4: 18–32	32.68 (22.35–44.77)	<0.0001
Adjusted*		
Loneliness Q1: 8–10	Ref	
Loneliness Q2: 11–13	2.45 (1.55–3.89)	0.0001
Loneliness Q3: 14–17	7.33 (4.81–11.17)	<0.0001
Loneliness Q4: 18–32	25.09 (16.63–37.84)	<0.0001

Note: Depression defined as moderate-to-severe depression *via* PHQ-9 scores of 10 or greater following standard PHQ-9 scoring criteria. Loneliness quartiles based on UCLA Loneliness Scale Short Form-8

Scores: Q1 = least lonely (scores 8–10), Q2 (scores 11–13), Q3 (scores 14–17), Q4 = most lonely (scores 18–32).

Abbreviations: Q: quartile; RR:relative risk; CI: confidence interval.

* Adjusted for age, gender, race, ethnicity, education, marital status, employment status, housing status, health insurance status, and social support.

**Table 4.** Univariable and multivariable associations of severity of loneliness with suicidal ideation.

	RR (95%CI)	*p*-value
Unadjusted		
Loneliness Q1: 8–10	Ref	
Loneliness Q2: 11–13	3.70 (1.62–8.44)	0.002
Loneliness Q3: 14–17	11.74 (5.45–25.28)	<0.0001
Loneliness Q4: 18–32	58.58 (27.81–123.41)	<0.0001
Adjusted*		
Loneliness Q1: 8–10	Ref	
Loneliness Q2: 11–13	4.03 (1.67–9.70)	0.002
Loneliness Q3: 14–17	11.87 (5.19–27.14)	<0.0001
Loneliness Q4: 18–32	44.31 (19.69–99.71)	<0.0001

Note: Suicidal ideation assessed *via* the ninth question of the PHQ-9. Loneliness quartiles based on UCLA Loneliness Scale Short Form-8

Scores: Q1 = least lonely (scores 8-10), Q2 (scores 11–13), Q3 (scores 14–17), Q4 = most lonely (scores 18-32).

Abbreviations: Q: quartile; RR: relative risk; CI: confidence interval.

* Adjusted for age, gender, race, ethnicity, education, marital status, employment status, housing status, health insurance status, and social support.
